# Rhabdomyosarcoma: Nose Presenting As Second Primary with Carcinoma Tongue

**DOI:** 10.22038/ijorl.2019.41435.2351

**Published:** 2020-03

**Authors:** Devanshu Kwatra, Poonam Sagar, Shailja Shukla

**Affiliations:** 1 *Department of Otolaryngology and Head and Neck Surgery, Lady Hardinge Medical College, New Delhi (India)-110001.*; 2 *Department of Pathology, Lady Hardinge Medical College, New Delhi (India)-110001.*

**Keywords:** Adult rhabdomyosarcoma, Nasal rhabdomyosarcoma, Rhabdomyosarcoma, Synchronous malignancy

## Abstract

**Introduction:**

Presence of two primary malignancies is rare and occurs in 3-5% of the cancer patients. As per our extensive internet research, this is the only reported case of a synchronous sino-nasal embryonal rhabdomyosarcoma with squamous cell carcinoma-tongue. The case report is important because of the rare diagnosis and the challenge we faced in the diagnosis and treatment of the patient because of the paucity of literature available on management adult rhabdomyosarcoma.

**Case Report:**

We present a very rare case of an adult male with a sino-nasal mass diagnosed to be an embryonal type rhabdomyosarcoma. The patient also had a moderately differentiated squamous cell carcinoma-tongue for the past 8 months. Radiological investigations were done to see the extent of the sino-nasal mass and the extent of tongue lesion, which was seen to be involving the base of the tongue. The patient was referred for chemoradiotherapy but succumbed to the disease after 2 weeks of treatment.

**Conclusion:**

Occurrence of rhabdomyosarcoma in synchronous malignancies is extremely rare as the most common first as well as second primary malignancy in a diagnosed case of head and neck cancer is squamous cell carcinoma. A multidisciplinary approach to the treatment of adult rhabdomyosarcoma has been recommended. The combined use of chemoradiotherapy and surgery has improved treatment in the recent past but RMS in adults is still a rare head and neck tumour that carries a poor prognosis despite aggressive therapy.

## Introduction

Presence of two primary malignancies is rare and occurs in 3-5 % of the cancer patients. In a case of head and neck cancer, the second primary tumour mostly develops in the head and neck region (1). In head and neck cancers, the most common index tumour seen is an oral cavity squamous cell carcinoma (SCC). The second primary is also of squamous cell variety in most cases (2). Here we present a rare case of a synchronous malignancy: squamous cell carcinoma-tongue, with a second primary: a sino-nasal embryonal rhabdomyosarcoma. This case report has substantial value, for it being a rare diagnosis and the challenge we faced in the diagnosis and treatment of the patient because of the paucity of literature available on management adult rhabdomyosarcoma.

## Case Report

**Fig 1:**
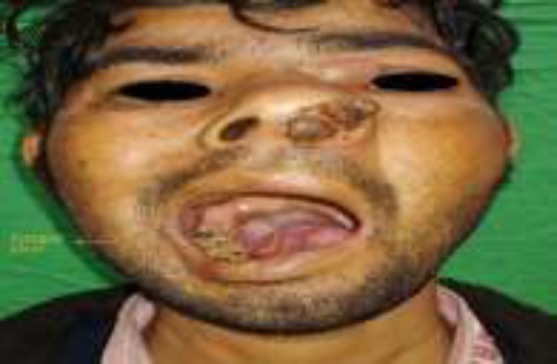
Clinical photograph of patient showing ulcer-infiltrative growth on the right lateral border of the tongue with a proliferative mass in the left nasal cavity along with left cheek swelling and left peri-orbital oedema

A 34-year-old male presented with a complaint of left nasal cavity mass, nasal obstruction for the past 6 months (Fig.1). 

**Fig 2A:**
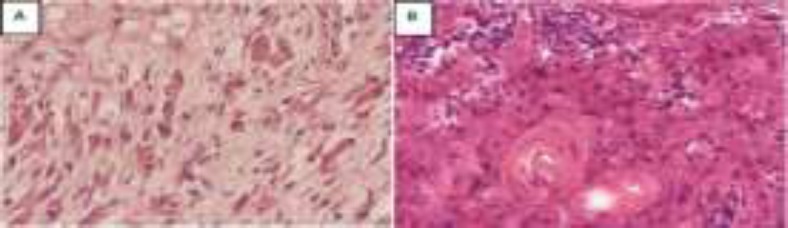
showing spindle-shaped elongated cells with acidophilic cytoplasm at 40x magnification which are characteristic of rhabdomyosarcomas. **B:** shows squamous cell carcinoma tongue with keratin pearls at 40x magnification

The patient also had a tongue ulcer on the left lateral aspect of the tongue for the past 8 months. The patient was already a diagnosed case of carcinoma tongue at the previous hospital 6 months back. The histopathology was moderately differentiated squamous cell carcinoma. The patient neglected tongue ulcer and did not take any treatment. There was no history of any substance abuse (Fig.2).

On examination, the oral cavity showed an ulcerative lesion over the left lateral border of the tongue (anterior two-thirds) of the size 3x1 cm. On palpation, ulcer base was indurated with induration extending posteriorly to involve left side tongue base. On nasal examination, widening of the nasal bridge along with left-sided periorbital swelling was present, more so in the region of the left medial canthus.

Reddish polypoidal mass was seen protruding from the left nostril which was covered with necrotic slough. The septum was pushed towards the opposite side. Contrast-enhanced computed tomography (CECT) of the nose and paranasal sinus showed heterogenous mass with mild enhancement completely filling the left nasal cavity with opacification of the left maxillary sinus and anteriorly extending out of the nasal cavity. Medially, the mass was causing bowing of the nasal septum towards the right. 

**Fig 3:**
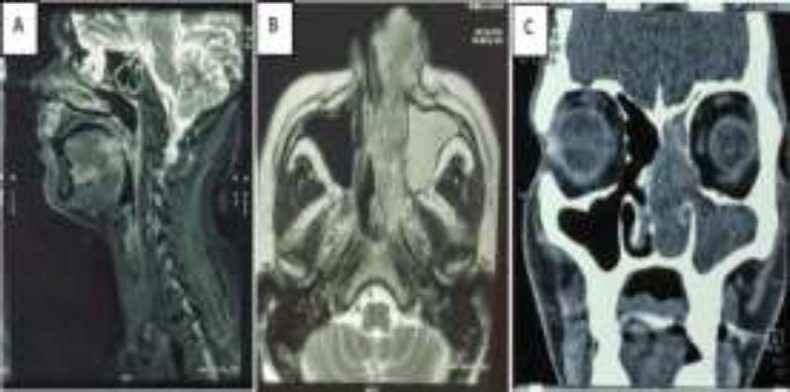
Radiological investigations. Image 3A showing Sagittal section MRI with a visible lesion involving the tongue base. Image 3B is an axial cut of MRI showing an irregular heterogeneously lobulated elongated soft tissue mass in the left nasal cavity extending onto deeper fascial planes at the medial canthus of the left orbit and into the left nasopharynx. Also visible is the mucosal thickening occupying the whole of the left maxillary sinus. Image 3C shows coronal section image of CECT Nose-Paranasal Sinus with evidence of a soft tissue mass filling the entire left nasal cavity. There is a widening of left osteo-meatal complex, bowing of septum to the right side due to mass effect of the lesion. The left side lamina papyracea appears to be eroded

Postero-superiorly, it was extending to the sphenoid sinus and causing widening of the on the left spheno-ethmoidal recess. Erosion of lamina papyracea was also seen on the left side Magnetic resonance imaging (MRI) face showed an irregular heterogeneously hypointense lobulated soft tissue mass (95 mm x31mm x48mm) in the left nasal cavity extending up to the left ethmoid sinuses superiorly, medial canthus of the left orbit antero-superiorly and into the left half of nasopharynx through left choana with associated mucosal thickening of the left maxillary and bilateral frontal sinuses. An enhancing lesion involving the base of tongue was also seen. (Fig.3).

Biopsy from nasal cavity mass showed elongated spindle-shaped cells with features of embryonal rhabdomyosarcoma (Fig.2). 

The biopsy sample was also subjected to immunohistochemistry with myogenin marker and the diagnosis was confirmed.

Owing to the pathology and the extent of the sino-nasal tumour, the case was discussed with the Department of Radiotherapy and a multimodality treatment was planned. The patient was referred for chemoradiotherapy (CRT) initially with surgical excision of the nasal mass later on kept as an option if required post-chemoradiotherapy. Unfortunately, the patient succumbed to the disease after 2 weeks of chemoradiotherapy due to the advanced stage of the disease and poor health.

## Discussion

Second primary malignancy (SPM) represents the leading cause of mortality in head and neck squamous cell carcinomas (HNSCC), responsible for approximately one-third of HNSCC deaths that is three times the deaths due to distant metastasis (2). 

Hong et al in 1990, based on the original criteria given by Warren and Gates in 1932, proposed the criteria for the definition of the second primary malignancy (SPM): the tumours have to be histologically certified as malignant; if they are of identical histological type, it must be an interval of at least three years between the two malignancies and/or presence of a distance of at least 2 cm of mucosa unchanged between the index tumour and the second primary tumour; it has to exclude the possibility that the second tumour to be the metastasis of the index tumor (1).

Notions of synchronous and metachronous cancers were first introduced by Warren and Gates in 1932, the concept of simultaneously occurring cancers being much more recent. Simultaneous tumours are defined as the tumours which develop in parallel with the initial tumour, synchronous, which develop within six months of the first malignancy and metachronous, which develops at least six months after the initial primary tumour development (3,4). The incidence of the second primary tumour is around 15 % for synchronous cancers and 4 per cent for metachronous cancers and this incidence increases with time, to approximately 30% at 10 years after the original tumour (5-7). The prognosis of synchronous tumours is worse as compared to metachronous tumours (4). The occurence of a secondary rhabdomyosarcoma after a primary squamous cell carcinoma is rare and several histogenic possibilities exist. To our knowledge, this is the first case of a rhabdomyosarcoma of sino-nasal cavity being secondary to a carcinoma primary of tongue, although, a few such cases with the primary affecting larynx have been mentioned in the literature. 

Antanova and Dormak described one such association of a 55 year old patient who had undergone subtotal laryngectomy (8).

There was topographic differentiation between the areas of carcinoma and rhabdomyosarcoma when histopathological examination of the resected specimen was done. There was no metastasis and thus the two malignancies were labelled as being separate from each other. The patient was well after 1 year of followup. Goldman et al in 1992 also described a similar case of synchronous occurrence of a rhabdomyosarcoma with carcinoma (9). They reported a case of squamous cell carcinoma of larynx with cervical nodal metastasis showing rhabdomyosarcomatous differentiation. 

However, the other affected cervical lymph node metastasis had shown squamous metaplasia except for the reported sample. They believed that the rhabdomyosarcomatous differentiation occurred at the site of squamous cell carcinoma metaplasia. According to them, the radiotherapy administered to the case before the rhabdomyosarcoma was identified might have played a role in its pathogenesis. In our case, the two malignancies were at separate locations, albeit affecting aerodigestive tract only. In both the above mentioned examples from past, the primary tumor was excised first and the secondary rhabdomyosarcoma was identified in the post-operative period for which subsequent chemoradiotherapy was given. We had directly sent the reported patient for chemoradiotherapy without surgical excision as the both the base of tongue carcinoma and the nasal rhabdomyosarcoma would not have benefited from surgical excision owing to the extent of lesion in the former and the histology in the latter. Sinonasal tumours often have a delayed presentation because of non-specific symptoms they present with, which often mimics symptoms of rhinosinusitis. In our case also, the patient when initially presented to another hospital with the complaint of nasal obstruction, treatment was started on lines of nasal polyposis even after getting a CT done. The patient was taking topical steroids for 3 months with no improvement before presenting to us. An MRI helped us to differentiate the mucosal changes in the maxillary sinus cavity from the nasal mass occupying whole of the left nasal cavity. This delay in diagnosis, coupled with the fact that the patient ignored the tongue lesion even after a diagnosis of malignancy was made blurs out the line between the appearance of 2 malignancies as to which came first.

Wurm et al did a retrospective analysis of 15 patients with RMS of the nose or paranasal sinuses treated between 1979 and 2000 (10).

The mean age of the affected patients in their study were 22 years and the embryonal type being the most common histological subtype. Thompson et al also did a similar retrospective analysis of 16 patients treated between 1992 to 2012 (11). They observed that sinonasal region is an unfavourable site for RMS with additional poor prognostic factors being age greater than 18 years and the alveolar histological subtype. RMS in adults usually affects non-head and neck regions. The four main histological subtypes of RMS are: embryonal, alveolar, pleomorphic and botyroid with the embryonal subtype being the most common (50-60%). It is important to recognize the correct histological subtype of RMS as the treatment protocol mentioned for adult RMS in literature is not as standardized as it is in case of children. RMS in adults has got a very poor prognosis with a median survival of 22 months without treatment (12).

## Conclusion

Adult RMS is a rare tumor and an even rarer occurrence in the form of a synchronous malignancy. Squamous cell carcinomas form the major chunk of head and neck cancers. SCC is the most common primary as well as second primary in head and neck malignancies. Typically, a second primary in case of a head and neck cancer would also be a squamous cell carcinoma but as in our case, a thorough and protocol based workup of the patient becomes necessary if one is suspecting a lesion at a site different from primary malignancy and metastasis is not being suspected or has been ruled out. A multidisciplinary approach to the treatment of adult RMS has been recommended because of the unresectabilty of the tumors in most cases and poor response after surgical excision alone. In the patient mentioned in our report, we could have gone for surgical excision first followed by chemoradiotherapy but the presence of another malignancy (the tongue carcinoma) which was unresectable made CRT a better option. Also, the paucity of literature available on the management of synchronous malignancies, one of which is a rhabdomyosarcoma posed difficulty in deciding the management protocol. The combined use of chemoradiotherapy and surgery has improved treatment in the recent past but RMS in adults is still a rare head and neck tumour that carries a poor prognosis despite aggressive therapy.
